# Learning from success: the main drivers of the maternal and newborn health transition in seven positive-outlier countries and implications for future policies and programmes

**DOI:** 10.1136/bmjgh-2023-012126

**Published:** 2024-05-06

**Authors:** Oona Maeve Renee Campbell, Agbessi Amouzou, Cauane Blumenberg, Ties Boerma, The Countdown to 2030 Exemplars Collaboration, Himanshu Bhushan

**Affiliations:** 1 Infectious Disease Epidemiology, London School of Hygiene and Tropical Medicine, Faculty of Epidemiology and Population Health, London, UK; 2 International Health, Johns Hopkins University Bloomberg School of Public Health, Baltimore, Maryland, USA; 3 International Center for Equity in Health, Universidade Federal de Pelotas, Pelotas, Brazil; 4 Community Health Sciences, University of Manitoba, Winnipeg, Manitoba, Canada

**Keywords:** Health policy, Child health, Health systems evaluation, Maternal health, Public Health

## Abstract

Currently, about 8% of deaths worldwide are maternal or neonatal deaths, or stillbirths. Maternal and neonatal mortality have been a focus of the Millenium Development Goals and the Sustainable Development Goals, and mortality levels have improved since the 1990s. We aim to answer two questions: What were the key drivers of maternal and neonatal mortality reductions seen in seven positive-outlier countries from 2000 to the present? How generalisable are the findings?

We identified positive-outlier countries with respect to maternal and neonatal mortality reduction since 2000. We selected seven, and synthesised experience to assess the contribution of the health sector to the mortality reduction, including the roles of access, uptake and quality of services, and of health system strengthening. We explored the wider context by examining the contribution of fertility declines, and the roles of socioeconomic and human development, particularly as they affected service use, the health system and fertility. We analysed government levers, namely policies and programmes implemented, investments in data and evidence, and political commitment and financing, and we examined international inputs. We contextualised these within a mortality transition framework.

We found that strategies evolved over time as the contacts women and neonates had with health services increased. The seven countries tended to align with global recommendations but could be distinguished in that they moved progressively towards implementing their goals and in scaling-up services, rather than merely adopting policies. Strategies differed by phase in the transition framework—one size did not fit all.

**WHAT IS ALREADY KNOWN ON THIS TOPIC**

- Most efforts to understand the drivers of mortality decline are either ecological comparisons of different countries at one time-point, multivariable analyses of individual-level risk factors for mortality, or short-term evaluations of selective interventions usually delivered as projects not government programmes; few factor in the effects of health system, health policy, or economic or human-development changes, and few address both maternal and neonatal outcomes.

**WHAT THIS STUDY ADDS**

- We examined a broad range of drivers of mortality decline for mothers and neonates, over 2–3 decades within positive-outlier countries, and interrogated distal, intermediate, and proximal determinants. We contextualised our countries within a transition framework, and showed that the strategies used evolved over time as contacts with health services increased.

**HOW THIS STUDY MIGHT AFFECT RESEARCH, PRACTICE OR POLICY**

- The approaches adopted by the seven countries tended to align with global recommendations, but could be distinguished by how countries progressively implemented their goals, rather than merely adopting policies. Strategies differed by phase—one size did not fit all. Deviations from global recommendations (eg, Bangladesh (relatively low institutional delivery) and Morocco (low antenatal care coverage and relatively few healthcare providers)) occurred, but these may not be associated with success in other settings; other deviations, (eg, Niger with high fertility) may foretell future stagnation in mortality decline.

## Introduction

 Each year, an estimated 0.3 million maternal deaths, 2 million stillbirths and 2.4 million neonatal deaths occur, comprising about 8% of deaths worldwide.^1^ Ambitious global and country goals have been set to reduce these deaths by 2030, but progress is often piecemeal.^2^ Weak health systems, low intervention coverage, social inequalities, and poor governance and implementation hinder many countries. Nevertheless, there are positive-outlier countries. Understanding the factors underpinning rapid progress in these countries can provide valuable learning for countries proceeding more slowly.

In this paper, we synthesised experience from across seven positive-outlier countries and assessed the contributions of non-health sectors and health sectors to maternal and neonatal mortality reduction. Detailed country-specific findings can be found in other papers in this supplement.^3^,^9^ We aimed to answer: What were the key drivers of maternal and neonatal mortality reductions seen in these countries from 2000 to the present? How generalisable are the findings?

### Approach, methods and frameworks

Positive-outlier countries were identified using estimates from the United Nations (UN) Interagency Group for Child Mortality Estimation and UN Maternal Mortality Estimation Interagency Group.^10 11^ Details of these and other methods are in online supplemental appendix 1, but in brief, candidate countries had larger-than-expected average annual rates of change between 2000 and 2017 for maternal mortality ratios (MMR), and between 2000 and 2019 for neonatal mortality rates (NMR), given their average annual growth rate in gross national income (GNI) per capita. Candidate countries needed to perform better than expected for maternal mortality, neonatal mortality or both. We included stillbirths in our analyses whenever feasible.

To select from among candidate countries, we assessed data availability (eg, health facility and household surveys) and considered feasibility, relevance and regional representation. We ultimately selected Ethiopia, Senegal, Niger, Morocco, Bangladesh, Nepal and India.

To understand national-level drivers, we used mixed-methods and worked in partnership with country experts in maternal or neonatal health, supported by researchers with method-specific or topic-specific expertise. We developed and used two frameworks: one which describes a comprehensive range of potential determinants^12^ and a second one which permits us to characterise our seven countries vis-a-vis others, and to guide suggestions for the future.^13^ We also explicitly considered whether countries had ‘learning health systems’.^14 15^

#### Determinants framework

The common determinants framework^12^ ensured we interrogated a comprehensive range of distal, intermediate and proximal determinants of maternal and neonatal health (MNH) and survival, and that we considered government levers for effecting change. This determinants framework was also a scaffold for integrating our mixed-method results and for systematically examining progress within the positive-outlier countries.

#### Transition framework

The maternal, stillbirth and neonatal mortality transition framework^13^ was used to benchmark changes in our seven countries compared with typical patterns observed in other countries at similar levels of mortality. The transition framework is premised on maternal, stillbirth and neonatal deaths having overlapping causes and common interventions, and has five phases, in which phase I has the highest maternal, stillbirth and neonatal mortality, and phase V the lowest. It is based on global estimates, data from historical populations and prospective population studies, and data from around 300 surveys. It provides comparative phase-specific cause-of-death patterns, fertility and socioeconomic indicators,^16^ data on health workforce and financing, contacts with services, and intervention coverage. Inequalities and neonatal mortality by place of birth are also examined by phase. The transition framework underlines that characteristics extant in one phase may be absent in another, and that strategies can change over time. We used the transition framework in this paper in part because we hypothesised that the drivers of mortality reductions in our seven countries were likely to be phase-dependent, and that we were unlikely to find ‘one-size-fits-all’ or ‘single’ determinants. We also liked that it could guide future action.

### Drivers of mortality reduction

**Figure 1:**
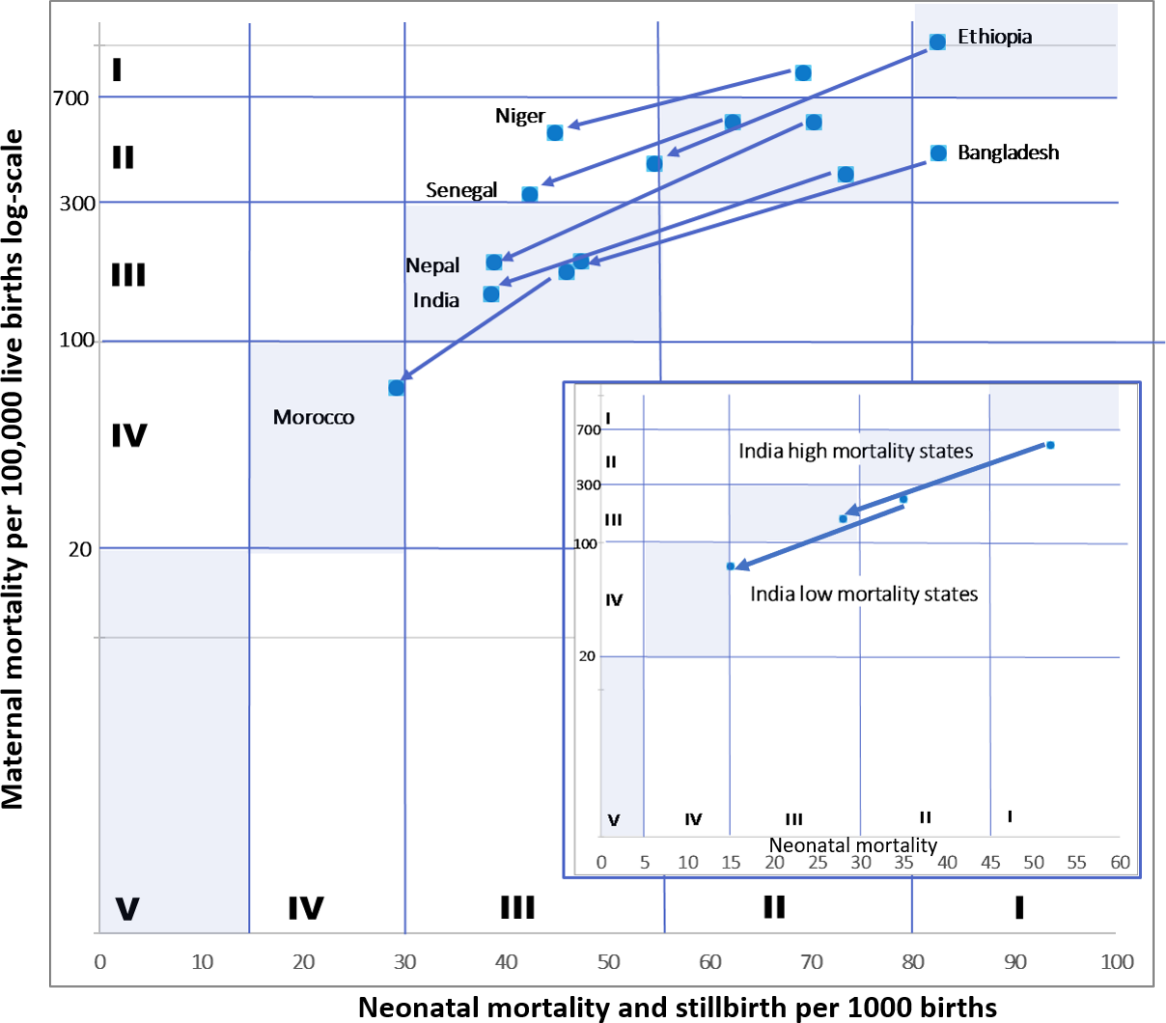
Mortality trajectories in the seven positive-outlier countries (2000–2017/2019) across the transition framework phases (log-scale maternal mortality ratio by sum of neonatal mortality and stillbirths per 1000 births). Inset is of two low-mortality Indian states and three high-mortality Indian states (log-scale maternal mortality ratio by neonatal mortality per 1000 live births).

Figure 1 shows mortality trajectories in the seven countries between 2000 and 2017/2019, across the transition framework phases; the inset shows higher-mortality and lower-mortality states in India. Ethiopia and Niger moved from phase I (highest mortality) to phase II. India’s higher mortality states and Bangladesh moved from phase I to phase III. India in its entirety and Nepal and Senegal moved from phase II to phase III, while India’s lower mortality states moved from phase II to phase IV. Morocco moved from phase III to phase IV. The causes-of-death shifted as predicted by the transition framework, from a greater predominance of infection and peripartum complications, to more underlying conditions of the mother and baby, but are not shown as they relied heavily on modelled data.

We grouped the drivers of the mortality reductions into four emerging themes, elaborated below: (1) increased contact with maternity services, (2) progress in quality of services, (3) fertility decline and safer abortion practices and (4) government levers, including strong commitment, resources and financing, and progressive implementation and learning.

#### Drivers of success: increased contact with maternity services

**Figure 2:**
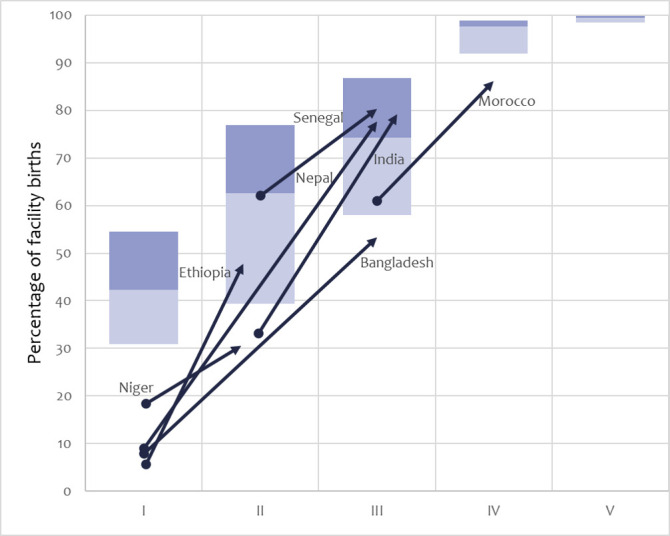
Trajectories of institutional births in the seven positive-outlier countries (nearest years to 2000–2019) across the transition framework phases. Boxes show the median and IQR of all countries by phase, according to household surveys 2000–2020.

Women in all seven countries increased contacts with childbirth, antenatal care (ANC) and, to a lesser extent, postnatal care (PNC) services. Figure 2 shows institutional childbirth coverage in the seven countries by transition framework phase from 2000 to 2019, against a backdrop of the median and interquartile range (IQR) for 300 surveys (2000–2020). The increases are spectacular. India, Nepal and Ethiopia moved from being below the IQR for their phase in 2000, to being within or above it by 2019. Bangladesh, Niger, Morocco also increased, but remained with lower institutional childbirth coverage than typical for their phase.

#### Intensity and timeliness of contact

Women also contacted maternity services earlier, more frequently, and for longer. ANC in the first trimester increased, as did the proportion of pregnant women with four or more ANC visits (ANC4+). Lengths-of-stay after childbirth increased in some countries, as did prompt PNC for women and neonates. In Nepal, for example, institutional delivery increased from 10% in 2001 to 78% in 2019; women were also more likely to use health facilities (ie, to go to hospitals) where emergency obstetric and neonatal care (EmONC) was available. This would have expedited emergency care if needed. ANC4+ rose from 14% to 78%, and a first ANC check within the first 4 months of pregnancy from 15% to 58%. PNC for mothers increased from 23% in 2006 to 58% in 2016 and for neonates from 32% in 2011 to 69% in 2019. Maternal and neonatal postnatal checks also happened sooner, with, for example, rising proportions occurring within 4 hours and within 1 hour of birth, respectively.^3^

#### How greater contact was achieved

Greater contact with maternity services was driven by distal determinants such as economic and human development, and by specific health-policy levers and strategies. The latter changed over time, but included demand generation, greater supply of facilities, and reductions in geographical and financial barriers. In the early transition phases, most of our seven countries expanded community outreach by community health workers (CHWs), including by using Accredited Social Health Activists (ASHAs) (India), community health extension workers (CHEWs) (Ethiopia and Bangladesh), female community health volunteers (FCHVs) and women’s groups (Nepal), bajenou gok (Senegal), and relais communautaires in health posts (Niger). Some CHWs provided preventive or treatment interventions directly (eg, family planning services), but most were used to generate demand for services by promoting or facilitating care-seeking.

On the supply side, countries expanded health infrastructure by increasing the number and geographical spread of health facilities, all aimed at improving access. For example, there were more integrated health centres I and II (Niger), growth in numbers of health posts, health centres and hospitals (Ethiopia), expanded Community Health Centres at subdistrict level (India), birthing centres placed in strategic areas 2+ hours away from primary health centres or district hospitals, and scale-up to ensure functioning hospitals and abortion services in all districts (Nepal), and more facilities and equitable implementation (Morocco). In Bangladesh, engagement with the (not-for-profit) private sector via community clinics began in 1996, providing an innovative model of public-and-private collaboration and community ownership. Bangladesh doubled its comprehensive EmONC facilities by the mid-2000s, and saw the construction of 18 000 community clinics and a rapid rise in private facilities offering normal deliveries. By 2016, all pregnant women were within an hour’s reach of a health facility for delivery care.

Investments to improve access were also made via maternity-waiting homes, and enhanced transport and referral systems. In Ethiopia, maternity-waiting homes were present in 56% of health centres and 18% of hospitals; in Niger, Mother-Child Referral Centres were constructed in each region. In Bangladesh, increased linkages were created between lower-level and higher-level facilities; in Morocco, ambulances, free referral between facilities levels, and a specific emergency-referral system in rural areas were implemented. In India, the 108-ambulance programme and call-centres ensured free transport for pregnant women and in Nepal, transport incentives were paid to women. Countries also worked to remove other financial obstacles, and to introduce pro-poor or geographically targeted schemes in part to address inequities in service use. Financing mechanisms are discussed below as a key government lever.

Distal, non-health sector drivers of better access included improvements in road systems and communication infrastructure. Governments in Morocco, Nepal, India and Ethiopia dramatically increased road network coverage; for example, Morocco’s road network increased by 41% from 32 049 km in 2000 to 45 240 km in 2021, with improvements targeted at rural areas. Greater wealth in all seven countries also expanded household ownership of mobile phones and motorised vehicles, which in turn would have facilitated access to, and use of, health facilities and improved interfacility linkages.

#### Drivers of success: progress in quality of care

A second theme to emerge was improved quality. Our seven countries not only increased contacts with maternity services, but also paralleled improvements in quality via enhanced facility readiness/capability, greater human resource skills and training, and increased coverage of preventive, detection and treatment interventions (the most proximate drivers of mortality reduction). This focus emerged particularly as countries moved into phases II and III.

#### Increasing use of services with greater readiness/capability and with more trained human resources

**Figure 3:**
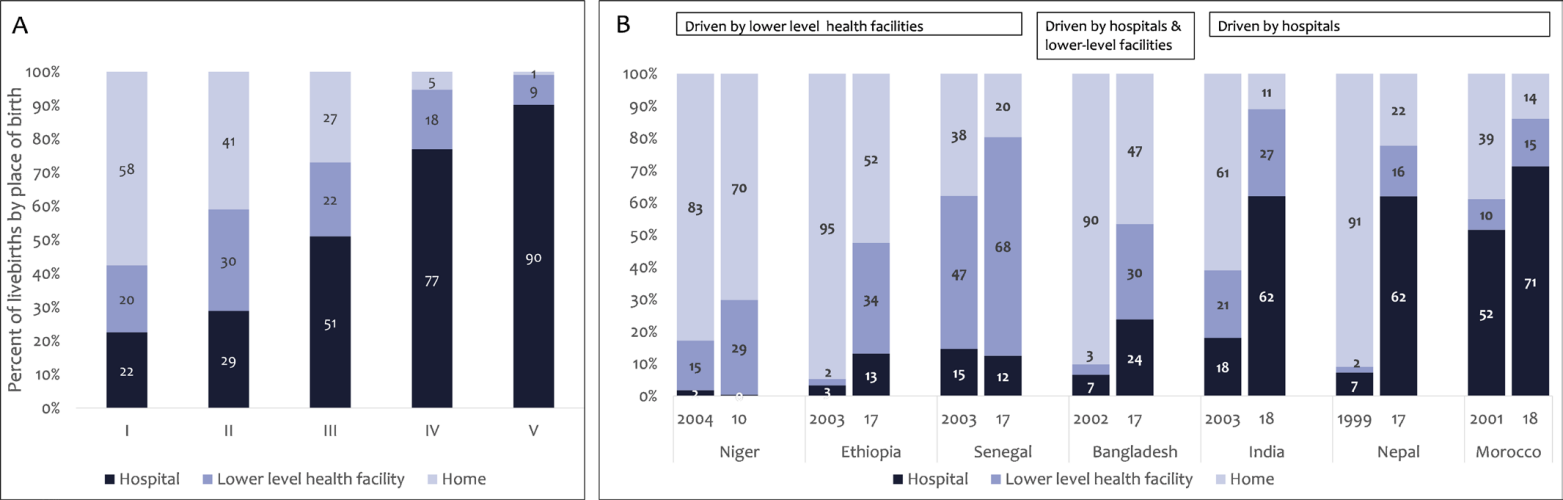
Place of birth (hospital, lower-level facility, home) (**A**) across the transition framework phases; and (**B**) the trajectories for the seven positive-outlier countries (nearest years to 2000–2019).

When women and babies contact health services, the level of health facility, who is there to provide care, and the available interventions (content-of-care) influence mortality outcomes. Figure 3 shows the level of health facility contacts for childbirth care, the period of highest risk^17^; it suggests lower mortality phases were most associated with hospital-focused strategies. Increases in institutional childbirth in phases I and II occurred primarily via lower-level facilities, but as countries approached universality of institutional childbirth, hospital deliveries began to predominate (phases III–IV–V). Increases in institutional childbirth in Niger, Ethiopia and Senegal were driven by births in lower-level facilities (I–II/early III); Bangladesh’s increase occurred in both hospitals and lower-level facilities. In India and Nepal, major increases led to 62% hospital births. Morocco started with 52% hospital births, and increased to 71%.

Health facilities’ abilities to provide emergency obstetric care (EmOC) (and to a lesser extent, neonatal care for small and sick newborns) also appeared to rise with time. Ethiopian health facilities increased their provision of signal functions between 2008 and 2016. Availability of essential medicines increased, including anticonvulsants (from 54% to 87%), antihypertensive medicines (from 78% to 92%), and oxytocics and prostaglandins (from 84% to 94%). Other functions started at high levels. India’s quality of Basic EmONC improved everywhere (after the National Rural Health Mission started), and Comprehensive EmONC capability expanded from 2012 onwards. Bangladesh expanded EmOC and qualified personnel to all 64 maternal and child welfare centres by 2003, and included specialised neonatal care units at district hospitals (SCANU programme). A substantial body of literature,^18 19^ and our own analyses, showed that lower-level facilities were generally less capable of providing Basic EmONC or routine care than hospitals, and that they frequently had poorer infrastructure and were more minimally staffed, with less-skilled cadres and few doctors.

Data available to us on human resources for health (HRH) tended to be of poor quality, and need to be interpreted cautiously. However, the transition framework suggests countries in phases I and II have a limited health workforce (4–8 doctors/nurse/midwives per 10 000 population), with 5–6 times more nurse/midwives than doctors. Moving to phase III and IV, there is a rapid increase (phase III—25 per 10 000 and phase IV—46 per 10 000), especially in doctors, so that the numbers of nurse/midwives become roughly double those of doctors. In our countries, Ethiopia had an impressive increase in its workforce, driven by nurses and midwives. It also implemented task-shifting strategies to respond to some stagnation in numbers. Nepal also increased its numbers of doctors, midwives and nurses from 6.8 in 2004 to 41.2 per 10 000 in 2019, and exceeded the 2014 International Labour Office recommendation of 34.5 per 10 000, and came close to the 2016 World Health Organization recommendation of 44 per 10 000. Nepal also made specific efforts with its workforce in remote areas, adopting transformative approaches such as training nurses to perform deliveries and anaesthesia. Indian data on HRH are problematic, but showed increases to phase-appropriate levels. Bangladesh’s increase was limited and almost entirely driven by increases in doctors, but it formalised midwifery as a distinct health cadre, and trained staff-nurses in delivery, and general doctors in EmOC and anaesthesia. Morocco increased its numbers of doctors, but illustrated that phase IV could be reached with a below-average workforce density.

#### Content/quality of services

Concomitant with increased contacts with services and improvements in health facility capability and HRH, our seven countries grew their ability to provide effective interventions (more content) when contacts with services occurred. Some interventions scaled up were ‘old’ (ie, countries had offered them for a long time (eg, tetanus-toxoid coverage)), while others were ‘new’, (ie, were initiated in the country during the time period under study, (eg, provision of abortion services in Nepal, or of neonatal resuscitation in Bangladesh).

Increases in ANC intervention coverage were easily tracked via population-based surveys, with women reporting more interventions being received in later years compared with 2000. All countries increased collection of blood samples for lab-testing during ANC; other ANC interventions (eg, blood pressure measurement, urine testing, iron supplementation and discussion of danger signs) also improved in most. For example, in Senegal, the percentage of pregnant women for whom a blood sample was taken rose from 6% to 59%, partly because ANC increased, but also because women attending ANC were more likely to get this intervention.

The content of childbirth care also appeared to improve over time when assessed via elements of routine childbirth care such as weighing of neonates, immediate breast feeding, hygienic cord-care and predischarge checks. For example, Nepal and Bangladesh introduced and scaled up chlorhexidine for cord care in health facilities and for home births.

**Figure 4:**
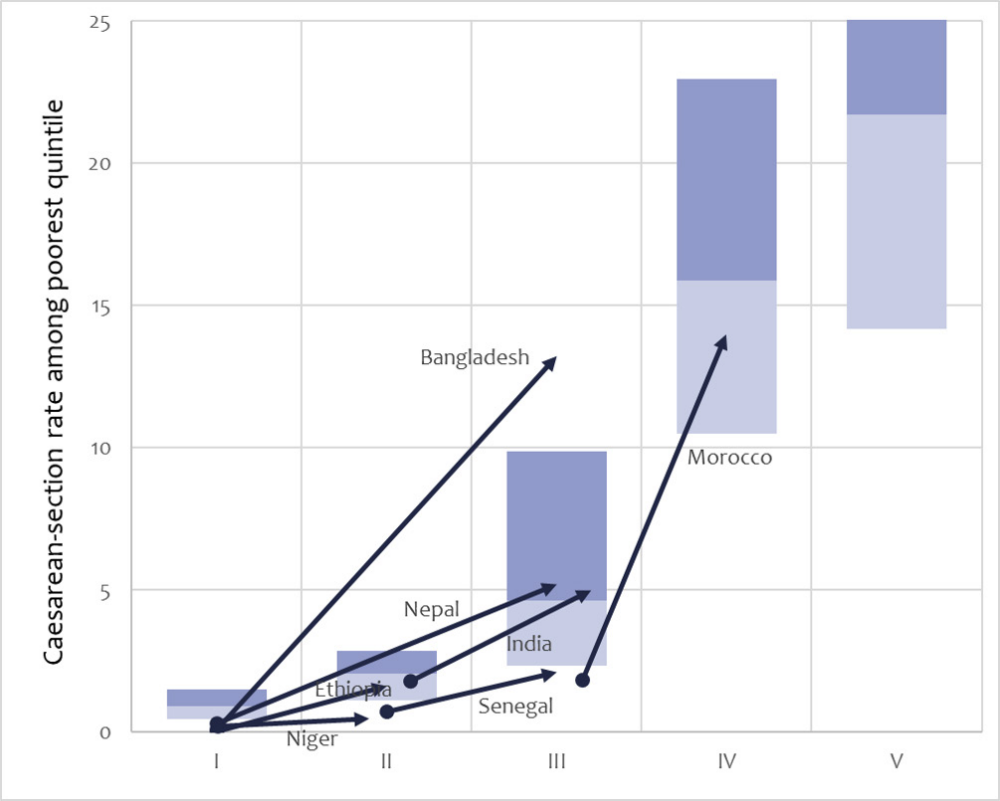
Caesarean-section rate trajectories among the poorest quintile in the seven positive-outlier countries (nearest years to 2000–2019) across the transition framework phases. Boxes show the median and IQR of all countries by phase, according to household surveys 2000–2020. In phase V, the upper bound of the IQR is truncated and should be 35%.

Caesarean section, a key life-saving intervention childbirth intervention for both women and their babies (although one which can be overused), was tracked via the caesarean section rate (%) among the poorest wealth quintile (figure 4). Caesarean section rates among the poorest were very low in phases I and II (<1%), with a major increase to almost 5% in phase III. Meeting the estimated need for caesarean section (at 10%) occurs only in phase IV, with a tripling of rates among the poorest. In 2000, caesarean section rates were extremely low in all seven countries. Increases in India and Nepal were facilitated by major upsurges in hospital births, but shortfalls in the estimated need for caesarean section remained among the poorest. Bangladesh was exceptional in its heavy reliance on caesarean section overall (33%), which ‘spilled over’ to the poorest (13%). Morocco also had adequate levels among the poorest, but began to show evidence of an ‘epidemic of caesarean sections’, as the rate in 2018 reached 21% overall, and 69% in the private sector. Caesarean sections (and hospital-level institutional births) among the poorest remained infrequent in Niger, Ethiopia and Senegal, even by 2020.

The Lives Saved Tool (LiST) was used to assess the contribution of individual interventions to maternal, stillbirth and neonatal mortality.^20^ We grouped LiST interventions into (1) periconceptual and family planning (2) antenatal (3) childbirth-related and (4) curative/postnatal. Across countries, we found childbirth-related interventions contributed to around 75% of maternal lives saved and around 68% of neonatal lives saved during 2000–2020. Results for individual countries are shown in other papers in this supplement. Periconceptual interventions, mostly family planning, contributed to around 20% to maternal lives saved, while curative/postnatal interventions contributed to around 26% of neonatal lives saved. Improvements in antenatal interventions made the smallest contributions, except for tetanus-toxoid vaccination in Ethiopia. In most countries, uterotonics for postpartum haemorrhage were the top life-saving maternal intervention, followed by magnesium sulfate for eclampsia, but these varied considerably by phase and by intervention coverage. For example, in Bangladesh, caesarean section was the most impactful intervention for both mothers and babies. In most other countries, the top contributing neonatal intervention was case management of sepsis/pneumonia (~20%), followed by caesarean section, neonatal resuscitation and three essential neonatal care interventions, again with considerable variation by phase and by intervention coverage.

#### How did countries improve the content of care?

Several of our seven countries developed explicit policies to improve quality of care across a range of areas. India emphasised quality in the National Health Mission RMNCH+A period (2012 onwards) via LaQshya and Dakshata initiatives, with training in facilities around competent and respectful maternity care; Niger had a targeted policy for improved quality of care starting in 2006, which led to increased breastfeeding, iron/folic-acid doubling, and mandatory management of abortion complications by health personnel. In practical terms, we found evidence of improvement across many elements of the health- system building blocks. The upgrading of facilities and the shift to higher-level facilities (hospitals) meant women and neonates were often in health facilities with better staffing, equipment and commodities. Upgrading HRH and training of staff in MNH skills featured in many countries, for example, with the training of doctors in Nepal to provide vacuum aspiration for abortion (2004) and then second trimester terminations (2007), followed by training of midwives in vacuum aspiration (2008), and training of doctors and midwives to provide medication abortion (2009). Countries also endeavoured to improve supply chains and ensure key interventions were added to essential drugs lists. Key levers for driving these changes included adoption of effective interventions, developing costed plans and using data to track intervention coverage; these are described in theme 4 below.

#### Drivers of success: fertility decline and safer abortion practices

**Figure 5:**
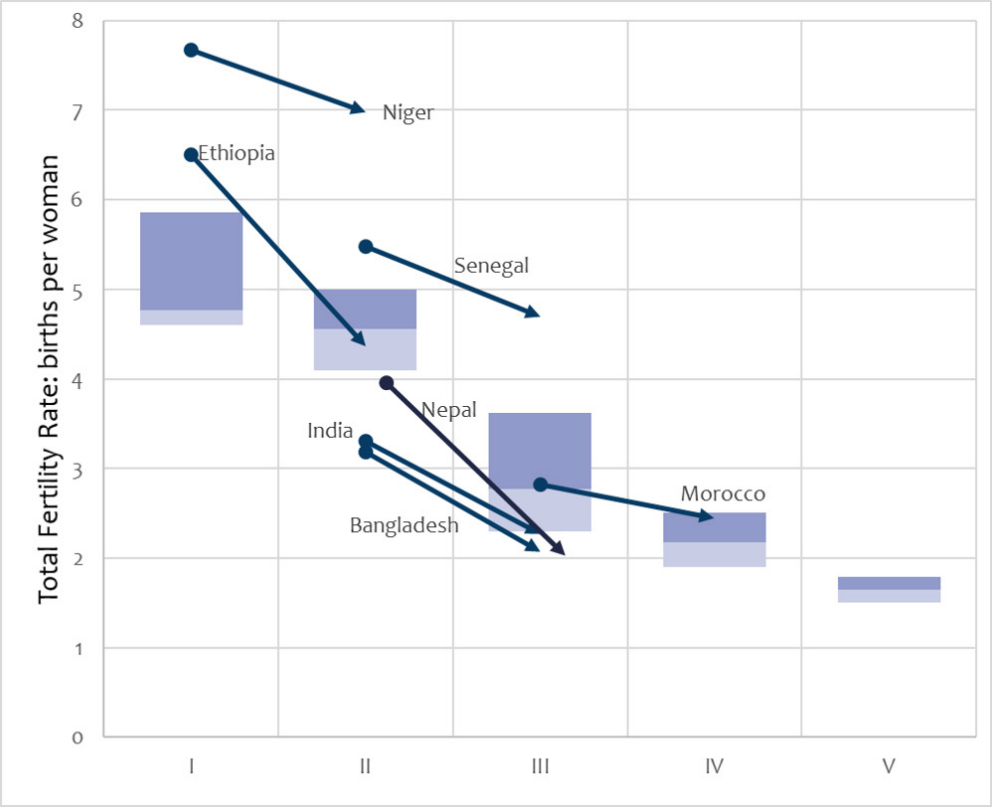
Total fertility rate trajectories in the seven positive-outlier countries (nearest years to 2000–2019), across the transition framework phases. Boxes show the median and IQR of all countries by phase, according to household surveys 2000–2020.

The drivers of success go beyond maternity and neonatal services and include fertility change and safer abortion. Figure 5 shows trends in the total fertility rate (TFR) in the seven countries (2000–2017) by transition phase, against a backdrop of the UN fertility estimates for 149 countries for the transition framework.

Bangladesh, India and Nepal had major fertility declines before 2000, and lower than typical TFR values for their phase, even as fertility continued to decline. Morocco followed this pattern to a lesser extent, although by 2017, its TFR was in the top half of the IQR for phase IV. Ethiopia also had a major decline from above the IQR for phase I, to a typical value for phase II. Niger was a major exception with high fertility and little decline.

Reduced fertility shifted age and parity patterns to more favourable, lower-risk mortality patterns. We quantified these via Jain’s decomposition approach,^21^ and estimated that fertility reduction contributed substantially to the decline in the MMR (12%–44%) and the NMR (16%–47%), except in Senegal and Niger where it contributed 6%–10% for MMR and 8%–12% for NMR. Reductions in birth rates, and the consequent levelling of numbers of births, would have paid a demographic dividend for MNH services, which would no longer have needed to grow at a rapid pace just to maintain coverage. Births were more likely to be wanted.

Why did fertility decline? The main proximate drivers are contraception, induced abortion, age at marriage (union), spousal separation and breastfeeding.^22^ We saw increases in contraceptive use in all countries, and more pronounced increases among rural and poorer women. Some countries also shifted to more effective contraceptive methods and improved supply chains. For example, Bangladesh produced its own family planning pharmaceuticals, and Senegal reinvigorated its family planning efforts in 2010 with the launch of FP2020 (now FP2030),^23^ and enhanced its contraceptive supply chains. In Bangladesh and India, fertility declines followed on from population policies and programmes that were instated decades before 2000.

India had permitted abortion up to 20 weeks since 1971 (expanded in 2021 to 24 weeks for some conditions) and Bangladesh had permitted menstrual regulation up to 12 weeks since 1979. In the last two decades, both Nepal and Ethiopia made their abortion laws more permissive and worked to ensure better access to abortion services. In addition, greater availability of medication abortion in most countries has meant even illegal services may be safer.^24^ Abortion laws in Morocco, Senegal and Niger remained restrictive.

Most countries increased their age at marriage, with concomitant declines in adolescent fertility. In Nepal and Morocco, substantial proportions of couples had men who migrated for work; this would have also reduced fertility.

Distal drivers, including improvements in women’s education and status and labour force participation, and shifting cultural norms around family size, are known contributors to fertility decline^25^; all countries showed improvements in women’s education, and many improved in the other areas as well. Reduced under-5 mortality is another predictor of reduced fertility,^26^ and our seven countries all improved in this area.

### Drivers of success: government levers, strong commitment, financing mechanism and progressive implementation

The determinants framework describes government levers for enacting improvements in health and in health systems as including ideas/policies, governance, laws/regulation, organisation, resources, financial incentives, and use of information and communication.^12^ Political prioritisation, initially of maternal health and later of neonatal health, played an essential role in many of the seven countries, and shaped the use of levers to improve MNH. In some countries, the political prioritisation and the policy and implementation processes centred around MNH or maternal health only, while in others it was ‘health-for-all’ or health equity. India, Bangladesh, and Morocco showed evidence of strong political interest in maternal mortality statistics. Starting around 2010, India and Bangladesh also became more interested in neonatal mortality statistics, a pattern seen in several countries. In some settings, champions and the media played an important role, for example, leadership by a specific health minister in Morocco. In India, the National Rural Health Mission gave MNH a central priority.

Legislation and regulation could also be significant. In 2003, a Supreme Court decision in Nepal changed abortion law to permit abortion on-demand up to 12 weeks’ gestation. A National Safe Abortion Policy was subsequently approved, and a Safe Abortion Advisory Committee established, leading to a series of steps to achieve gradual scale up and expansion of services over a 15-year time-frame. Nepal’s Ministry of Health regulations were changed to allow task-shifting.

Strategic planning, a system of developing and reviewing health strategies, policies, and plans, is conducted in many countries.^27^ The seven positive-outlier countries implemented most policies and plans, and modified them in response to progress made—exemplifying ‘learning health systems’. Nepal passed a Skilled Birth Attendant Policy in 2006 that outlined short-term, medium-term and long-term approaches to improving the quality of health workers’ training, and worked systematically to achieve this, testing and adapting policies as it went along. The high-level political leadership championed health, rather than MNH per se, but a relatively effective Civil Service was adept at carrying out implementation plans. Costed national health plans also helped ensure budgets covered commodities needed.

Our seven countries also worked to remove financial obstacles and to introduce pro-poor or geographically targeted schemes, in part to address inequities in service use. Financial protection mechanisms appeared to have only been partially successful, as rich-poor coverage gaps widened in most countries. Targeted efforts were sometimes directed at mothers and babies, or were part of an orientation to improve health equity overall. Progress among the poorest women was driven by demand generation and access improvement, removal of financial barriers and incentives in some countries. User-fee removal programmes for maternity care were implemented in Niger, Senegal, Ethiopia and Morocco, and conditional cash transfers were used via Janani Suraksha Yojana (JSY)/Janani Shishu Suraksha Karyakaram (JSSK) in India; Maternal Health Voucher Scheme in Bangladesh and the Aama programme in Nepal. The latter also used geographically differentiated incentives to give women in the mountains greater transport incentives than those in the Terai. In one instance at least, a legal lever was used to address financial access: in Nepal in 2009, a Supreme Court case, Lakshmi Dhikta v. Nepal, ruled access to safe abortion was a human right regardless of ability to pay, and resulted in the first annual budget to implement ‘Free Safe Abortion Services’ in 2016.

Another shared feature in several of our countries was their effective use of data. For example, Bangladesh measured maternal mortality through large surveys, and had multiple and regularly conducted Demographic and Health Surveys (DHS) or Multiple Indicator Cluster Surveys (MICS) since 1990. Senegal used ‘continuous’ DHS and Service Provision Assessment (SPA) surveys. Nepal produced detailed annual reports includingata from Health Management Information Systems (HMIS) and other statistics and programme evaluations. Ethiopia had an extensive learning phase to generate evidence for its health extension programme.

We also found evidence that countries kept abreast of the evidence on effective interventions, using international and national research. Nepal for example often trialled interventions (such as women’s groups,^28^ or vitamin A^29^ or chlorhexidine^30^) and then used this evidence to scale up. It then tracked uptake of the interventions carefully, often measuring them via HMIS or by adding relevant questions to DHS surveys and discussing them in annual reports. Key informants described how they then sought to address any shortfalls via revised strategies or targeted implementation. Bangladesh and Nepal worked well with funders, international agencies and non-governmental organizations to target specific areas of need.

## Discussion

Our analysis, particularly use of the transition framework, helped us understand and position the changes seen in seven positive-outlier countries. All countries shifted phases, with higher-than-expected declines in mortality given their GNI per capita; they showed causes-of-death as predicted by the transition framework.

The LiST analyses showed that the greatest contributors to lives saved between 2000 and 2020 were interventions for childbirth care, substantially overshadowing interventions in the antenatal or postnatal periods. Crucially, it was the configuration of services, and the increased contacts with health facilities, particularly for childbirth, that enabled countries to deliver the preventive or treatment interventions that reduced mortality. In early phases, and in sub-Saharan Africa, institutional births were predominantly in lower-level health facilities. A big question is how much this approach can reduce mortality. If lower-level facilities are not equipped or staffed to provide life-saving care, the benefits of health facility contact may be minimal. A focus on hospital births or on upgrading lower-level facilities and ensuring effective referral for women and babies who cannot be managed at lower levels are the main options. Nepal, India and Morocco have opted for the former (with over 60% hospital births) but even they may need to exceed 80% hospital births for phases IV and V to meet their Sustainable Development Goal targets. Other questions include how to improve access to hospitals given the current configuration of healthcare in these countries? Should countries prioritise building hospitals (or transforming health centres into hospitals) over lower level facilities or direct all deliveries to hospitals? What would be the effect on other aspects of healthcare provided at lower-level facilities such as child or adolescent health or family planning?

Some countries appeared to be outliers in their configuration of services. In Bangladesh, for example, this included high levels of home births, reliance on the private sector, and high levels of caesarean section, even for the poorest. Others seemed to achieve success without having outstanding levels of any given parameter. For example, Morocco achieved low mortality without having very high levels of ANC or PNC, but had good levels of hospital delivery. Nor did it have very high densities of doctors and nurse-midwives.

We showed that increased contacts with health services, coupled with increased content (improved quality) once women came to services, meant spectacular increases in coverage of interventions at the population level. A comparison of chlorhexidine coverage illustrates how place-of-birth affects intervention coverage at the population level. Bangladesh^31^ and Nepal^32^ implemented chlorhexidine for cord-care for both home and institutional births. Both countries achieved 33% coverage within homes, and 77% and 69% within health facilities for Bangladesh and Nepal respectively. Yet because Bangladesh had low levels of institutional births (53%), and Nepal had high levels (78%), estimated population level coverage of chlorhexidine for cord care was higher in Nepal (61%) than Bangladesh (56%).

The poor benefited substantially, but in many areas inequalities in coverage did not reduce. In several countries, caesarean section among the poorest reached 5%, the minimum level necessary, but not Niger, Senegal or Ethiopia.^33^ Reaching all disadvantaged women and newborns will be critical for countries to reach phases IV and V.

Jain’s decomposition and LiST suggested fertility decline and family planning were important contributors, although the extent estimated by the two models differed because LiST only assessed the contraception component of fertility reduction. We also noted Niger’s outlier status in remaining at a high TFR. Our transition framework would predict this could inhibit further mortality reductions.

Finally, we reviewed the government levers used to enact improvements. Political prioritisation played an essential role, shaping the adoption of policies, strategies, laws and financing to improve MNH. Strategic planning, and learning health system featured strongly in several countries. Countries also worked to remove financial obstacles. While out-of-pocket expenditures increased in many countries, it is possible that household MNH expenditure did not, because of fee-removal and cash-transfer financing policies protected these services. Unfortunately, we had no data with which to verify or refute this hypothesis.

Another paper in this volume addresses measurement challenges.^34^ In brief, challenges included poor data on policies; short time series; a lack of availability of some anticipated sources (such as SPAs) because COVID-19 interrupted field work. We were unable to build a single model to pick out the most salient drivers, in part because of data gaps and because some drivers, such as policies, were implemented at national level. Our time period of interest preceded the COVID-19 pandemic and did not assess the impacts of climate change. On the other hand, strengths include the utility of subnational analyses in some countries (eg, India), the positive-health framing and the long-time periods considered. We also gained enormously from the insights of team members with decades-long experience of MNH in-country.

## Conclusion

In conclusion, our research built a rich body of evidence on the most salient drivers of success in seven positive-outlier countries over the past 2–3 decades. The determinants framework provided a comprehensive scaffold for interrogating the drivers of maternal, neonatal and stillbirth mortality declines. However, another approach was needed to analyse and interpret the vast amount of data. We found the transition framework was a useful tool to interpret the key dimensions of the progress of countries, including the current situation, and that it could also guide discussions on past progress, current situations and future strategies in all. In summary, we found evidence that most countries made concerted efforts to address many of the issues highlighted in global strategy setting, and tried to ensure improvements in many of the health-system building blocks. A distinctive feature was that many of these countries exhibited learning health systems which enabled them to collect relevant data, reflect on them, and make adaptive improvements over time.

## Supplementary material

10.1136/bmjgh-2023-012126 online supplemental file 1
