# Supplementary material for: Learning from success: the main drivers of the maternal and newborn health transition in seven positive-outlier countries and implications for future policies and programmes
Source: BMJ Glob Health. 2024 May 6;9(Suppl 2):e012126. doi: 10.1136/bmjgh-2023-012126 (PMC11085707; doi:10.1136/bmjgh-2023-012126)
Supplement: online supplemental file 1 [file bmjgh-2023-012126supp001.pdf]

Appendix 1. Methods

Selection of positive outlier countries:

We selected positive-outlier countries using IGME data sources (UN Interagency Group for Child Mortality Estimation)(1) and MMEIG (UN Maternal Mortality Estimation Interagency Group)(2), although we also ran sensitivity analyses with Institute for Health Metrics and Evaluation (IHME) data. We excluded former countries (e.g. Yugoslavia), or countries which at the start of the period (2000) had missing data, had already achieved low mortality (NMR < 12 and MMR < 70), had a population of <2 million, or were high- or upper-middle income. We then identified countries with larger than expected average annual rates of change between 2000 and 2017 for maternal mortality and between 2000 and 2019 for neonatal mortality, given the compound annual growth rate in the Gross National Income (GNI) per capita (Atlas Method, current US$) obtained from the World Bank.(3) Candidate countries performed better than the expected relationship between mortality change and GNI per-capita change for either maternal mortality, or neonatal mortality, or both (see Supplementary Figure 1).

Supplementary Figure 1. Countries plotted by the actual versus expected average annual rates of change given Gross National Income (GNI) per capita for maternal mortality ratio (MMR) in 2017 and neonatal mortality rate (NMR) in 2019*

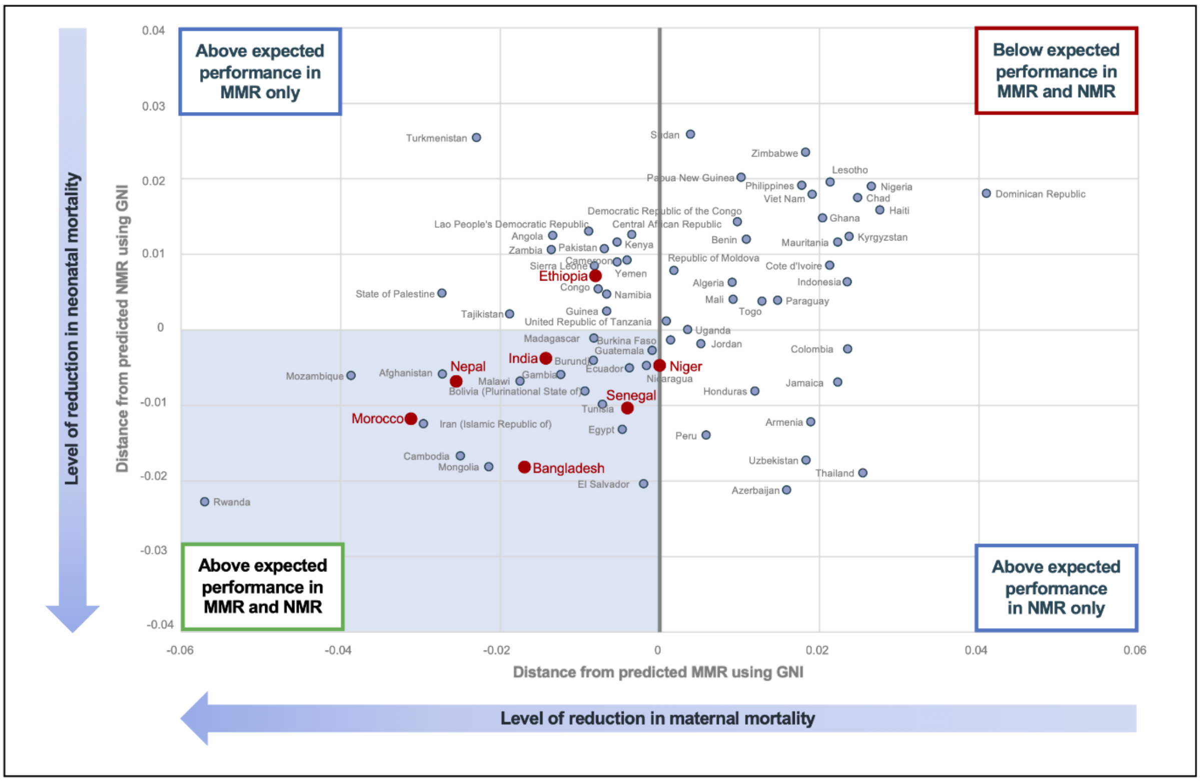

*Candidate countries were primarily in the lower left quadrant. Selected countries are show in red.

To select from among candidate countries, we assessed data availability (e.g. facility and household surveys). We excluded countries with conflict (e.g. Yemen) or where we could not speak the language (e.g. Angola) and considered other facets of feasibility, relevance, and regional representation. We excluded over-studied countries (e.g., Rwanda) and those with limited generalizability of findings (e.g. Kazakhstan). We prioritized countries where we had good contacts, and ultimately selected Ethiopia, Senegal, Niger, Morocco, Bangladesh, Nepal, and India; in India we explicitly selected 9 higher and 10

lower mortality states. Higher mortality states (49% of India's population) were: Bihar, Chhattisgarh, Jharkhand, Madhya Pradesh, Odisha, Rajasthan, Uttar Pradesh, Uttarakhand (comprising Empowered Action Group), and Assam. Lower mortality states (47% of India's population) were: Andhra Pradesh, Gujarat, Haryana, Karnataka, Kerala, Maharashtra, Punjab, Tamil Nadu, Telangana, and West Bengal.

Ways of working in countries:

National research institutions in the seven countries led in-country research (Supplementary Table 1). Where possible, national teams included investigators with 20+ years' experience in maternal or neonatal health programming or research. Other team members worked in or across countries, bringing a range of disciplinary or subject-specific knowledge (e.g., in health-systems or equity analyses). We obtained ethics approval from a local institution in each country and from at least one other institution from among the University of Manitoba, Johns Hopkins University, or London School of Hygiene & Tropical Medicine

Data and analytic approaches:

In-country research was conducted from mid-2020 to May 2022. Methods included reviews of published and grey literature, use of UN indicator databases, and further analysis of existing data from nationally-representative facility and household surveys, and from health management information systems (HMIS).

In many settings, facility data came from Service Provision Assessments (SPA) or Service Availability and Readiness Assessments (SARA)) surveys. Household surveys came from the Demographic and Health Surveys (DHS), Multiple-Indicator Cluster Surveys (MICS) or PAPFAM (PanArab Project for Family Health). Causes of neonatal death came from the World Health Organization's Maternal Child Epidemiology Estimation Group (WHO/MCEE), and from country reports and surveys where these were available. The proportion of infants born protected from tetanus infection were based on WHO/UNICEF annual estimates.

Quantitative analyses of HMIS data, and of DHS, MICS, SARA, and SPA surveys, included cross-tabulations and regression analyses, and used Stata(4), SPSS,(5) or R.(6) When looking at temporal trends, we used surveys closest in time to 2000 and to 2020.

We assessed the contribution of fertility decline using the approach proposed by Jain and colleagues and the contribution of individual interventions to maternal, stillbirth, and neonatal mortality using the Lives Saved Tool (LiST).(7) We calculated ANCq, a ten-point scale capturing coverage and quality of antenatal care (ANC) as described by Arroyave and colleagues.(8) We also experimented with a decomposition approach(9) and an approach comparing regional clusters, but found neither approach to be very illuminating, and so dropped them from our reporting.

For our health-system analyses, we performed desk-reviews of planning and policy documents, and developed policy timelines; in all countries, we conducted national- and district-level key informant interviews on the health-system context and organization, policy and programme development, and challenges and successes. Thematic analyses were carried out these interviews using a range of software, including nVivo(10).

Supplementary Table 1: Exemplars Partners

Country partners	
India	International Institute for Population Studies, Maharashtra, and National Health Systems Resource Centre, New Delhi
Ethiopia	Ethiopia Public Health Institute (EPHI) Addis Ababa, and Ministry of Health Addis Ababa, Ethiopia
Bangladesh	ICDDR,B, Dhaka
Nepal	South Asian Institute for Policy Analysis and Leadership (Saipal), Kathmandu
Morocco	National School of Public Health and Mohammed V Rabat University, Rabat, with the Institute of Tropical Medicine, Antwerp, Belgium
Senegal	Medical Research Council Unit, The Gambia, Dakar
Niger	the Institut National de la Statistique, Niamey
Countdown to 2030 for Women's, Children's, and Adolescents' Health	
Brazil	International Center for Equity in Health Federal University of Pelotas
Kenya	African Population and Health Research Centre
Canada	Institute for Global Public Health of University of Manitoba
USA	Bloomberg School of Public Health of Johns Hopkins University
UK	London School of Hygiene & Tropical Medicine

References

1. United Nations Inter-agency Group for Child Mortality Estimation (IGME). Child mortality and stillbirth estimates [Available from: https://childmortality.org/.

2. United Nations Maternal Mortality Estimation Inter-agency Group (MMEIG). Inter-agency child and maternal mortality estimates [Available from: https://www.un.org/development/desa/pd/content/inter-agency-child-and-maternal-mortality-estimates.

3. The World Bank. Indicators [Available from: https://data.worldbank.org/indicator.

4. StataCorp. Stata Statistical Software: Release 17. College Station, TX: StataCorp LLC; 2021.

5. IBM SPSS Statistics for Windows. Version 27.0. Armonk, New York: IBM Corp; 2020.

6. R Core Team. R: A language and environment for statistical computing. Vienna, Asutria: R Foundation for Statistical Computing; 2013 [Available from: http://www.R-project.org/

7. Walker N, Tam Y, Friberg IK. Overview of the lives saved tool (LiST). BMC Public Health. 2013;13(1):1-6.

8. Arroyave L, Saad-Haddad G, Victora CG, Barros A. 40 An antenatal care indicator based on contact with services and content developed for 63 countries. International Journal of Epidemiology. 2021;50(Supplement_1):dyab168. 040.

9. Vaivada T, Akseer N, Akseer S, Somaskandan A, Stefopulos M, Bhutta ZA. Stunting in childhood: an overview of global burden, trends, determinants, and drivers of decline. The American journal of clinical nutrition. 2020;112(Supplement_2):777S-91S.

10. QSR International Pty Ltd. NVivo qualitative data analysis software (Version 12) 2018 [Available from: https://www.qsrinternational.com/nvivo-qualitative-data-analysis-software/home.
